# Multi-Parameter Relief Map from High-Resolution DEMs: A Case Study of Mudstone Badland

**DOI:** 10.3390/ijerph16071109

**Published:** 2019-03-28

**Authors:** Hone-Jay Chu, Yi-Chin Chen, Muhammad Zeeshan Ali, Bernhard Höfle

**Affiliations:** 1 Department of Geomatics, National Cheng Kung University, Tainan City 701, Taiwan; zeeshanktk1992@yahoo.com; 2 Department of Geography, National Changhua University of Education, Changhua City 50007, Taiwan; yichinchen@cc.ncue.edu.tw; 3 Institute of Geography, Heidelberg University, Heidelberg 69047, Germany; hoefle@uni-heidelberg.de

**Keywords:** multi-parameter relief map, red relief, 3P relief map, DEM, badland

## Abstract

Topographic parameters of high-resolution digital elevation models (DEMs) with meter to sub-meter spatial resolution, such as slope, curvature, openness, and wetness index, show the spatial properties and surface characterizations of terrains. The multi-parameter relief map, including two-parameter (2P) or three-parameter (3P) information, can visualize the topographic slope and terrain concavities and convexities in the hue, saturation, and value (HSV) color system. Various combinations of the topographic parameters can be used in the relief map, for instance, using wetness index for upstream representation. In particular, 3P relief maps are integrated from three critical topographic parameters including wetness or aspect, slope, and openness data. This study offers an effective way to explore the combination of topographic parameters in visualizing terrain features using multi-parameter relief maps in badlands and in showing the effects of smoothing and parameter selection. The multi-parameter relief images of high-resolution DEMs clearly show micro-topographic features, e.g., popcorn-like morphology and rill.

## 1. Introduction

Digital elevation models (DEMs) are widely applied in landscape and evolutionary ecology [1], soil mapping [2], landslide susceptibility [3], terrain deformation [4], tectonic/landslide fault discrimination [5], geo-hydrological processes [6,7], and archaeology [8]. Several technologies such as LiDAR, aerial photogrammetry, or radar interferometry are used to derive high-resolution DEM data [9]. In high-resolution DEMs with meter to sub-meter spatial resolution [9], topographic parameters such as slope, curvature, aspect, openness, and wetness index represent spatial properties and surface characterizations of the terrains and landscapes for landslide identification [10], erosion susceptibility [11], geo-archaeology [12], soil classification [13], and soil water retention prediction [14].

High-resolution DEMs produce accurate and detailed representations of terrains. However, the topographic parameter map is the most popular approach for terrain representation/visualization and the interpretation of topographic information [11]. Different sets of topographic parameters were used to describe and identify terrain features. The simultaneous visualization of multiple topographical parameters is a critical challenge. In the red, green, blue, and alpha (RGBA) color system, the approach is for overlapping multiple layers of the RGB images controlling alpha. Moreover, topographical parameters can be represented in the hue, saturation, value (HSV) color system [15]. For example, eight aspect categories were mapped with an orderly progression of colors around the hue circle. The lightness sequences approximated relief shading. Three slope categories were mapped with differences in saturation, and near-flat slopes were mapped with gray. Moreover, the red relief image map is also used in the HSV color system and considers the two topographic parameters of (i) slope and (ii) openness [16,17]. In this study, the various combinations of two topographic parameters are used in two-parameter (2P) relief maps. The 2P relief map is the extension of the red relief map. The slope of the DEM is the major choice for the relief map. The second parameter of the map, such as openness or total curvature, is then selected. Multi-parameter relief maps are developed to include two or three nearly-independent topographic parameters. Furthermore, the three-parameter (3P) relief map is also applied in the HSV color system using three topographical factors, e.g., elevation, slope, and openness. Slope and curvature-based information are two major datasets in the multi-parameter relief map. However, curvature is the second derivative of the DEM. The differentiation of DEM increases the image noise. The noisy curvature directly affects the multi-parameter relief image and should not be used as input. Therefore, smoothing or de-noising is important for high-resolution DEMs, especially for higher-order derivative images. The smoothing techniques from image filtering are effective in removing noise [18]. Median filtering is one of the famous smoothing techniques. In this study, median filtering was used for smoothing the curvature image.

This study offers an effective way to explore the combination of topographic parameters to show terrain features using multi-parameter relief maps in the badland area. The multi-parameter (2P or 3P) relief map provides combinations of topographic information such as slope, curvature, wetness index, or hillshading. Moreover, the smoothing process of DEM derivatives is used to remove topographical noise for mapping, especially in the very high-resolution DEM. High-resolution relief maps with the slope and openness/plan curvature/total curvature is applied to identify the micro-topographic features of the mudstone badlands.

## 2. Materials and Study Area

**Figure 1:**
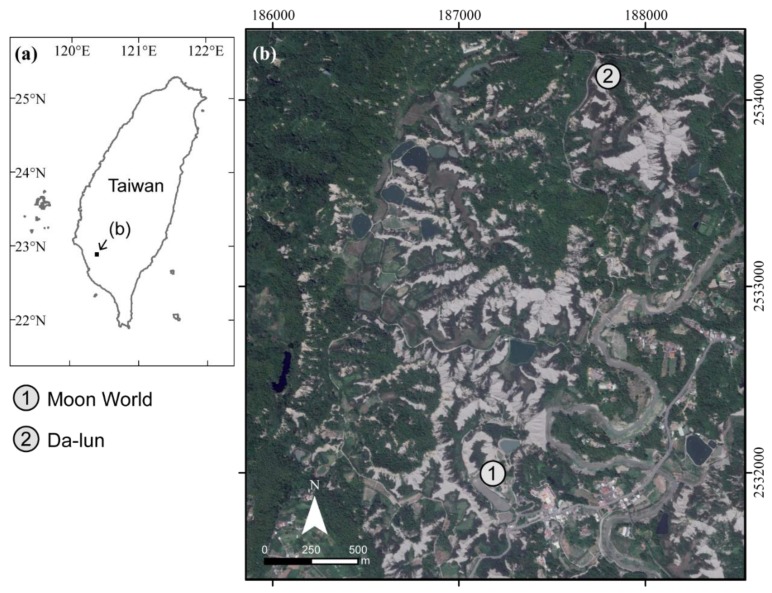
Location of the study area in southern Taiwan (image source: Google Earth).

The study area is a mudstone badland region which is located upstream of the Erren River in Kaohsiung, south of Taiwan (Figure 1). The lithological formation in the study site is the Plio-Pleistocene Gutingkeng Formation with gray mudstone interbedded with some sandstone [19]. The area’s distinctive mudstone is comprised of clay and silt fractions with weak rock strength [19]. The climate in this area is tropical monsoon, with an average rainfall of 2030 mm/year measured from the Gutingkeng meteorological station [20]. Over 90% of the annual rainfall occurs during the wet season (May to October). Weak rock strength combined with intensive rainfall causes rapid erosion rates of 65.0–80.7 mm/year, which is about 10–13 times higher than the average erosion rate in Taiwan [21], and barren mudstone hillslopes with densified gullies have thus developed. Each time it rains, the surface absorbs most of the moisture and expands as a result. When the sun comes out, the surface becomes cracked because of its dryness. Repeated wetting and drying cycles compromise the surface integrity of the mudstone [21,22,23] and cause a popcorn-like morphology, with many cracks extending to depths of a few centimeters [24]. Downpours cause erosion, thus forming exposed ridges and gullies with sparse vegetation cover.

Two mudstone badland sites were selected as the study area. In Moon World (Area I), the average elevation was 41 m above sea level whereas the average slope was 38.3 degrees. The study area, which comprised 0.45 ha, was a result of the mudstone features being weakly lithified into aquitards. Area II was located upstream of Area I. The study area, which comprised 0.18 ha, was characterized by its barren and rugged topography and densified gullies that had developed. The average elevation was 96 m above sea level whereas the average slope was 44.9 degrees.

The spatial resolutions of the two datasets of the DEM were 1 m (Area I) and 1 cm (Area II) in this study. The high-resolution DEMs were derived based on a UAV-SfM (structure from motion) sequence. The multi-view UAV photography was taken using a DJI Phantom 4 Pro, a commercial quadcopter equipped with a non-metric camera with a flying height of approximately 20 m above ground on 10 February 2018 (Area II) and 21 April 2016 (Area I). The Pix4DMapper, a commercial photogrammetry software, was used to perform image matching, extract point clouds, and generate DEMs and ortho-images. To correct the position for the whole process, a set of ground control points was surveyed with real-time kinematic GNSS. Based on the standard workflow of the UAV-SfM, the output DEMs had a ground sampling distance (GSD) of 5 mm and a minor root mean square error of 2.4 mm in vertical direction compared with 58 individual checkpoints (Area II). Photogrammetric image processing enabled the creation of high-resolution DEMs. All images were processed using photogrammetric software which involved interior orientation, exterior orientation, and aerial triangulation.

## 3. Methods

**Figure 2:**
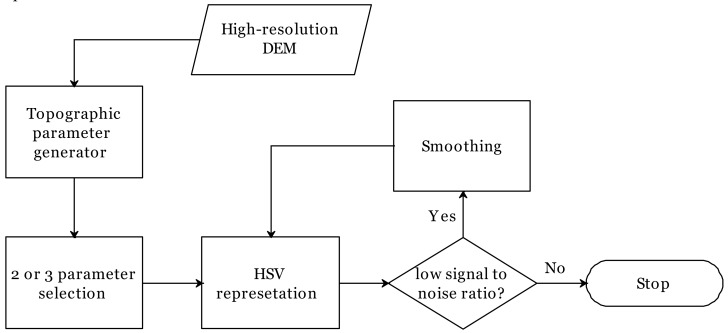
Flowchart of multi-parameter (two-parameter (2P) or three-parameter (3P)) relief mapping in high-resolution digital elevation models (DEMs).

The approach for multiple topographic parameter visualization for the 2P or 3P relief map contained four main parts including (1) topographic parameter generation, (2) topographic parameter selection, (3) the smoothing of DEM derivatives, and (4) representation/visualization (Figure 2).

### 3.1. Topographic Parameter Generator

The generator was used to calculate the slope, aspect, openness, hillshade, wetness index, profile curvature, plan curvature, and total curvature for the DEMs [16,25,26]. The slope and curvature were the first and second derivations of the DEM [27]. In this study, the calculation of the curvatures was based on the definitions of Zevenbergen and Thorne [28]. Numerically, the gradient and Laplacian were the first and second derivatives of the DEM [29]. The openness incorporates the terrain line-of-sight principle from zenith and nadir angles along eight azimuths [16]. Openness is not subject to directional bias [30]. Terrain openness is a measure of the features of the valley (negative value of openness) and ridge (positive value of openness).

In addition, the wetness index is a function of both the slope and the upstream contributing area per unit width orthogonal to the flow direction [31]. The index was designed to identify hydrological flow paths for hydrological processes [31].

### 3.2. Topographic Parameter Selection

Slope was the first choice of key feature for terrain topographic analysis. Further, the openness or plan curvature/total curvature was selected for the analysis. Furthermore, wetness index, aspect, and hillshade were selected for further terrain topographic analysis.

### 3.3. Smoothing of DEM Derivatives

Image noise is easily generated from the curvature process in high-resolution DEMs. The noisy curvature directly affects the appearance of the 2P relief image and should not be used as input. In this study, the median filter [32] was applied for the smoothing of the curvature image. The moving window size was 7 × 7 pixels.

### 3.4. Representation/Visualization

In the 2P relief map, using the red hue, the topographic slope was shown as the saturation value and the openness/curvature was shown as the brightness [17]. In the 3P relief map, the elevation or the aspect or wetness index was further applied as the hue value, combining it with the slope and openness.

## 4. Result and Discussion

### 4.1. 2P Relief Map Based on Slope and Curvature

**Figure 3:**
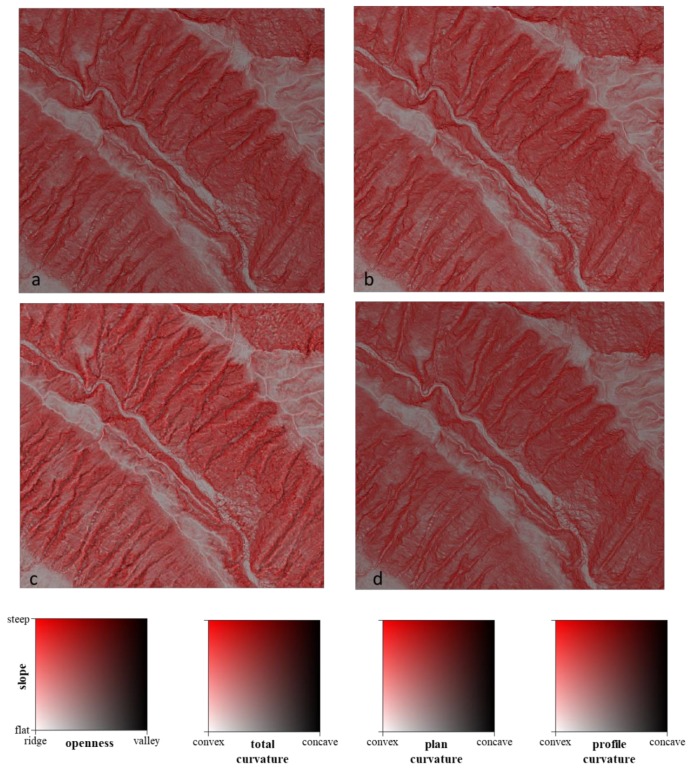
(**a**) 2P relief maps with slope and openness, (**b**) slope and total curvature, (**c**) slope and plan curvature, and (**d**) slope and profile curvature.

Figure 3 shows the 2P relief maps considering slope- and curvature-based features, such as openness, total curvature, plan curvature, and profile curvature (Area II). Similar results (Figure 3a–c) for the ridges are shown as a white color, whereas the valleys are displayed as a black/dark color. Steep slopes are visualized as bright red, and flat surfaces have a gray color. However, valleys (rill) cannot be found in a black or dark color based on the 2P relief with the slope–profile curvature (Figure 3d).

The correlation between openness and total curvature (correlation coefficient *r* = 0.98) is higher than the correlation between openness and plan curvature (correlation coefficient *r* = 0.72). However, the correlation between openness and profile curvature is negative (correlation coefficient *r* = −0.87). The 2P relief map implies that it can be used for the identification of valleys (rill) and ridges when based on slope and the features of openness, total curvature, and especially plan curvature. The highlighted relief features distinguish the highest and lowest areas of features without any horizontal displacement [30].

### 4.2. Smoothing Effect

**Figure 4:**
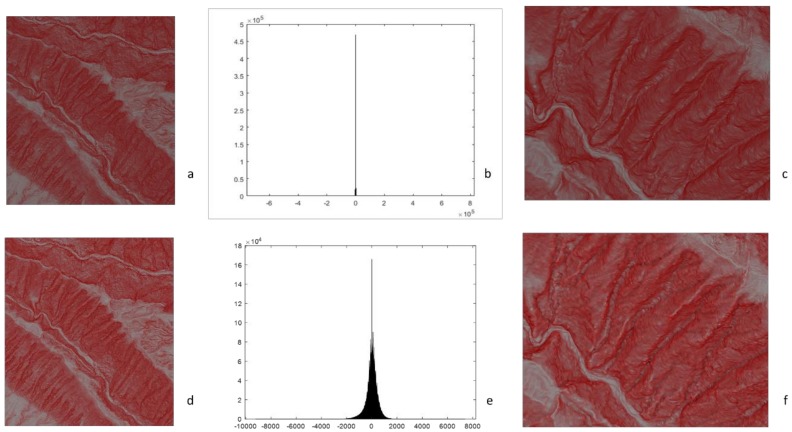
2P relief maps, histograms and detailed comparisons (**a**–**c**) without filtering of the plan curvature and (**d**–**f**) with filtering of the plan curvature.

Figure 4 shows the 2P relief maps in the high-resolution DEMs before and after smoothing, as seen in Figure 4a,d, and their histograms of curvatures, seen in Figure 4b,e. Originally, the curvature image contains noise and shows a low signal-to-noise ratio (SNR). After filtering/smoothing, the range of the curvature reduces to between 8000 and −10,000.

Figure 4c,f also shows a detailed comparison of plan curvature without and with filtering. In Figure 4e, without smoothing, the valleys look like the ridges, and the valleys in the dark areas of the 2P relief image are not clear. In Figure 4f, after smoothing, the valleys are shown in dark colors. Combing the smooth curvature image, the valley can be identified clearly in the dark areas of the red relief image. As the high-order derivative of the DEM increases the image noise, smoothing can reduce the image noise if the terrain is highly varied with space, especially in high-resolution DEMs. In the 2P relief image of the high-resolution DEM, smoothing is the important step to reduce the noise of the high-order derivation of DEMs.

### 4.3. Topographic Parameter Selection of 2P Relief Image

Table 1 shows the correlation coefficients of slope and openness with the topographic parameters (Area I). The slope has a low correlation with the curvatures. The openness is highly correlated to total curvature and plan curvature with *r* = 0.98 and 0.90, respectively. Therefore, the openness in the red relief map can be replaced by plan curvature and total curvature. In the 2P relief maps, this terrain visualization was based on slope-based and curvature-based information for the HSV system.

**Figure 5:**
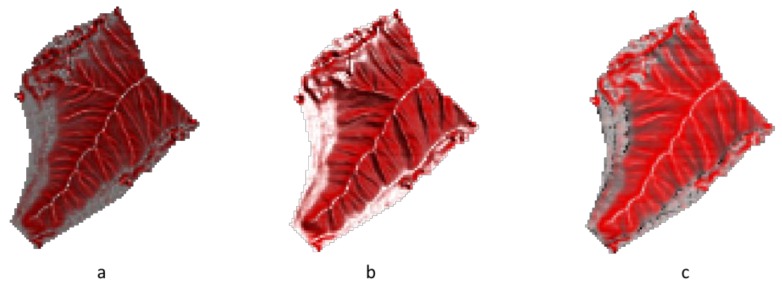
2P relief maps of (**a**) slope–openness, (**b**) slope–aspect, and (**c**) slope–wetness.

For visualization, the second parameter of the 2P relief map is flexible. For example, hillshade or wetness index can be used as the second parameter. Figure 5 shows the 2P relief map with slope–openness, slope–aspect, and slope–wetness in Area I. This result shows that the wetness index can easily be used to identify the downstream and upstream areas of the watershed. The upstream area is indicated by a light grey color, whereas the downstream is assigned a black color. In addition, two-directional hillshade is a simple and fast way to produce a fine visualization compared to more complex and better multidirectional hillshading methods [33]. Using the 2P relief map, the way forward is to investigate the applicability of the topographic parameters to function as a basis for automatic feature classification [26,30].

### 4.4. Three Topographic Parameters on a Map

**Figure 6:**
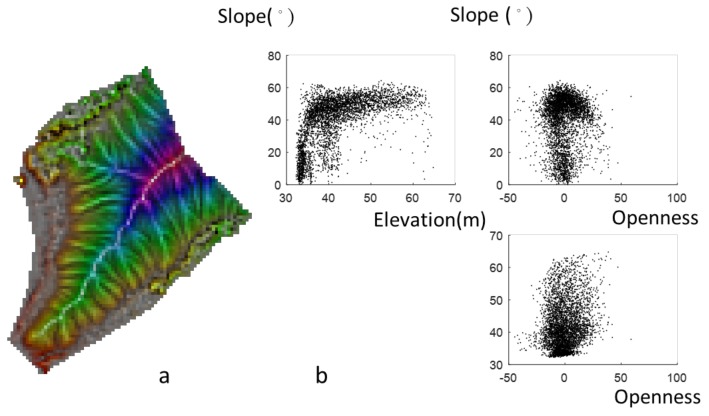
(**a**) 3P relief map and (**b**) scatter plots of elevation, slope, and openness.

**Figure 7:**
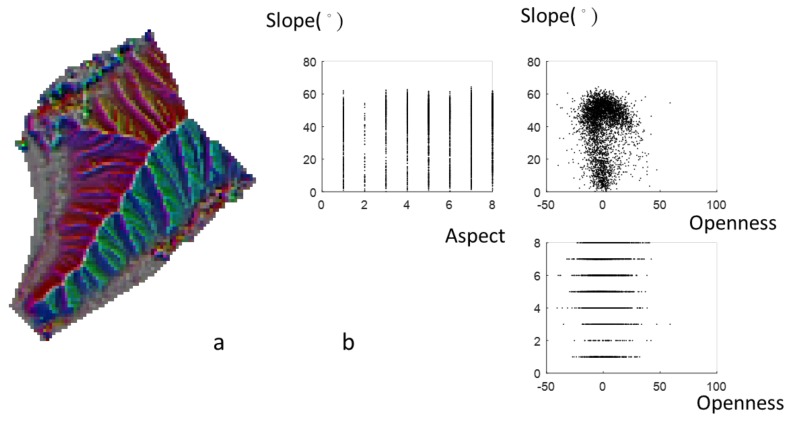
(**a**) 3P relief map and (**b**) scatter plots of aspect, slope, and openness.

**Figure 8:**
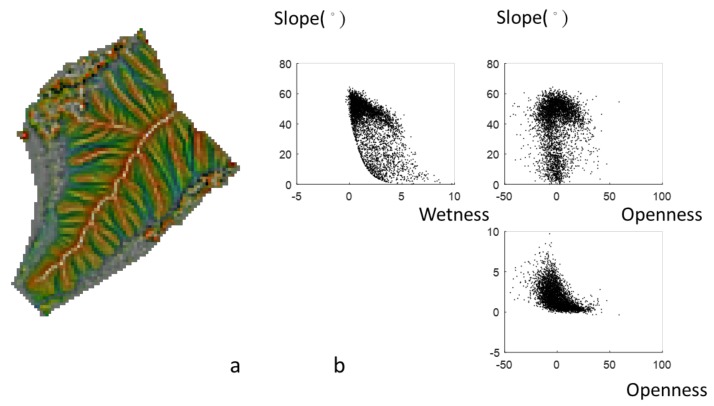
(**a**) 3P relief map and (**b**) scatter plots of wetness, slope, and openness.

The combination of three topographic parameters on a map is a practical application. Figure 6a shows the 3P relief map (Area I). The color map provides the information of the slope, curvature, and elevation, and it implies that Area I is an individual hill of a small size. The area is mostly covered by steep slopes, especially in the valley and ridge, as seen in Figure 6b. Figure 7a provides not only slope and curvature information but also aspect information. Aspect is not directly related to the slope and openness, as seen in Table 1. Figure 7b shows the scatter plots for uniform aspect distributions, and the slopes are between 0° and 60°. Figure 8a shows the combination of three parameters in a 3P relief map, namely slope, curvature, and wetness with true color tones. This 3P relief map obviously demonstrates the ridges and valleys in the badland. The wetness is useful to identify the upstream and the downstream areas of the watershed with lower and higher wetness. Zero wetness index occurs at the ridge (zero slope and high value of openness), as seen in Figure 8b. The 3P relief map showing the wetness can be easily linked to the hydrological processes and soil erosion. Moreover, approaches to directly measure erosion rates are costly and time-consuming (surveying methods, e.g., erosion pins, profilometers, levelling, and tracers [34]), whereas topographic analysis can facilitate the prediction of erosion rates rapidly and over large spatial extents [35]. However, now UAV remote sensing can derive terrain models and then estimate the soil erosion. Compared with traditional methods, UAV remote sensing is a highly practical and cost-effective approach for determining the spatiotemporal variation of soil erosion [36,37].

### 4.5. Mapping Micro-Topography

**Figure 9:**
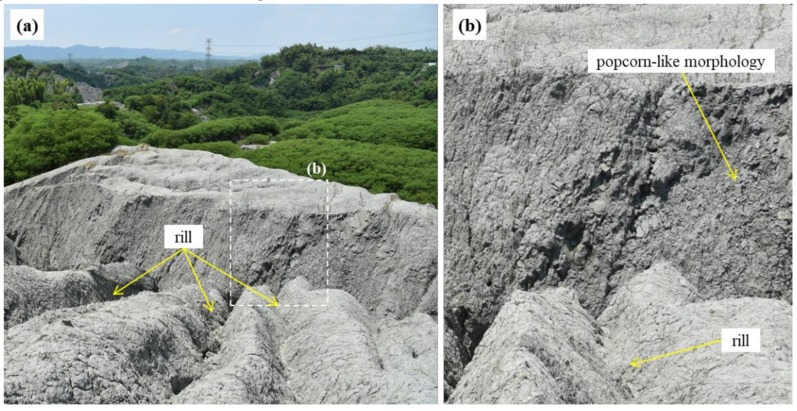
Popcorn-like morphology (**a**) and rill on steep hillslopes (**b**).

**Figure 10:**
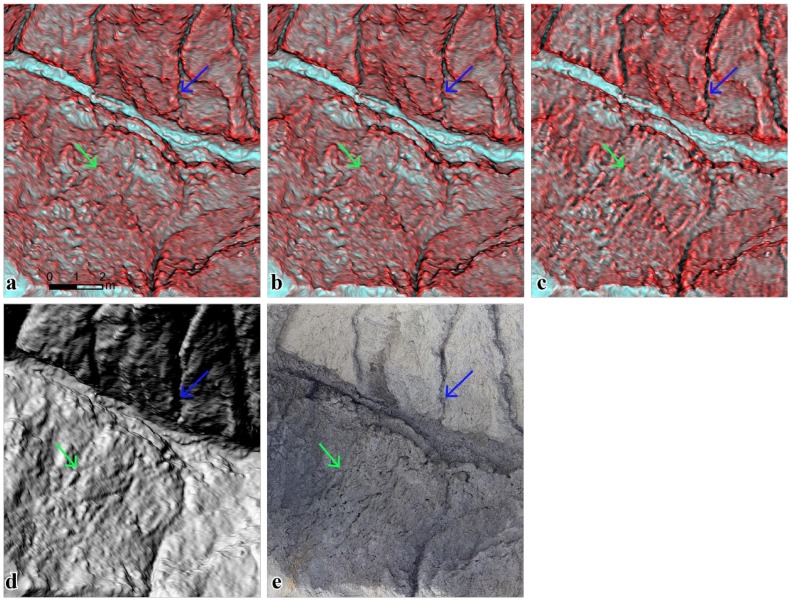
(**a**) Comparison of the 2P relief maps with slope–openness, (**b**) with slope–total curvature, (**c**) with slope–plan curvature, (**d**) hillshade map, and (**e**) ortho-image (popcorn-like morphology: green arrow and rill: blue arrow).

From the geomorphological perspective, there are four major erosion mechanisms present in mudstone badland landscapes, namely sheet erosion, rill erosion, collapsing, and subsurface piping [38]. The erosion mechanisms can be recognized by mapping the micro-topographic features. For example, collapsing is prone to occur on the mudstone surface with popcorn-like morphology and densified shrinkage-cracks [21,23], as shown in Figure 9. Figure 10 shows the 2P relief maps, hillshade map, and ortho-image on the mudstone badland region of Area II. The red relief maps with slope–openness, seen in Figure 10a, and slope–total curvature, seen in Figure 10b, clearly show the densified shrinkage-cracks and popcorn-like morphology, which are demonstrated by the green arrows in Figure 10. Moreover, the slope–plan curvature map, shown in Figure 10c, shows clear rills but exaggerated features and horizontal stripes in popcorn-like morphology. Furthermore, rill erosion is concentrated water flow eroding the surrounding materials on a micro-valley (<1 m in width), which is indicated by the blue arrows in Figure 10.

The micro-topography of rill is highlighted and recognizable on the 2P relief maps in Figure 10a–c, especially Figure 10c showing the slope–plan curvature. In contrast, in the hillshade maps shown in Figure 10d, the topography of rill is blocked by dark shadows and are difficult to recognize on a steep valley and on the aspects opposite to the light azimuth angle. Consequently, the high-resolution 2P relief maps with slope–openness, seen in Figure 10a, and slope–total curvature, seen in Figure 10b, are useful to show the micro-topographic features for identifying the major erosion processes in the mudstone badland. Rill features are more highlighted in the 2P relief map with slope–plan curvature, as seen in Figure 10c.

## 5. Conclusions

The effectiveness of terrain visualization and mapping was explored based on multi-parameter (2P and 3P) relief maps. The micro-features or micro-topography in badlands can be detected using this high-resolution DEM visualization. Various combinations of the topographic parameters (slope, openness, total curvature, plan curvature, and profile curvature) can be applied in the 2P and 3P relief maps. Wetness index, aspect, and hillshade were selected for further terrain visualization, especially wetness index for stream representation. Moreover, 3P relief maps were clearly displayed using three topographic parameters, e.g., elevation or aspect, slope, and curvature.

DEM differentiation (second derivative of DEMs for curvature) increases the image noise in high-resolution DEMs. The noisy curvature directly affects the 2P relief image and should not be used as input. Therefore, following the smoothing of the curvature image, the micro-topographic features (rill and popcorn-like morphology) can be obviously shown in the multi-parameter relief image of the high-resolution DEMs. This approach is highly practical and cost-effective for environmental monitoring and determining landscape spatial patterns.

## Figures and Tables

**Table 1:** Correlation coefficients of topographical parameters with slope and openness.

Parameters	Slope	Openness
Aspect	0.10	0.29
Total curvature	<0.001	0.98
Plan curvature	0.001	0.90
Profile curvature	0.002	−0.77
Wetness index	−0.61	−0.54
Hillshade	−0.71	−0.03
Openness	0.03	-
